# Supplementary figures and images for: TRPM4 regulates hilar mossy cell loss in temporal lobe epilepsy
Source: BMC Biol. 2023 Apr 26;21:96. doi: 10.1186/s12915-023-01604-3 (PMC10134545; doi:10.1186/s12915-023-01604-3)

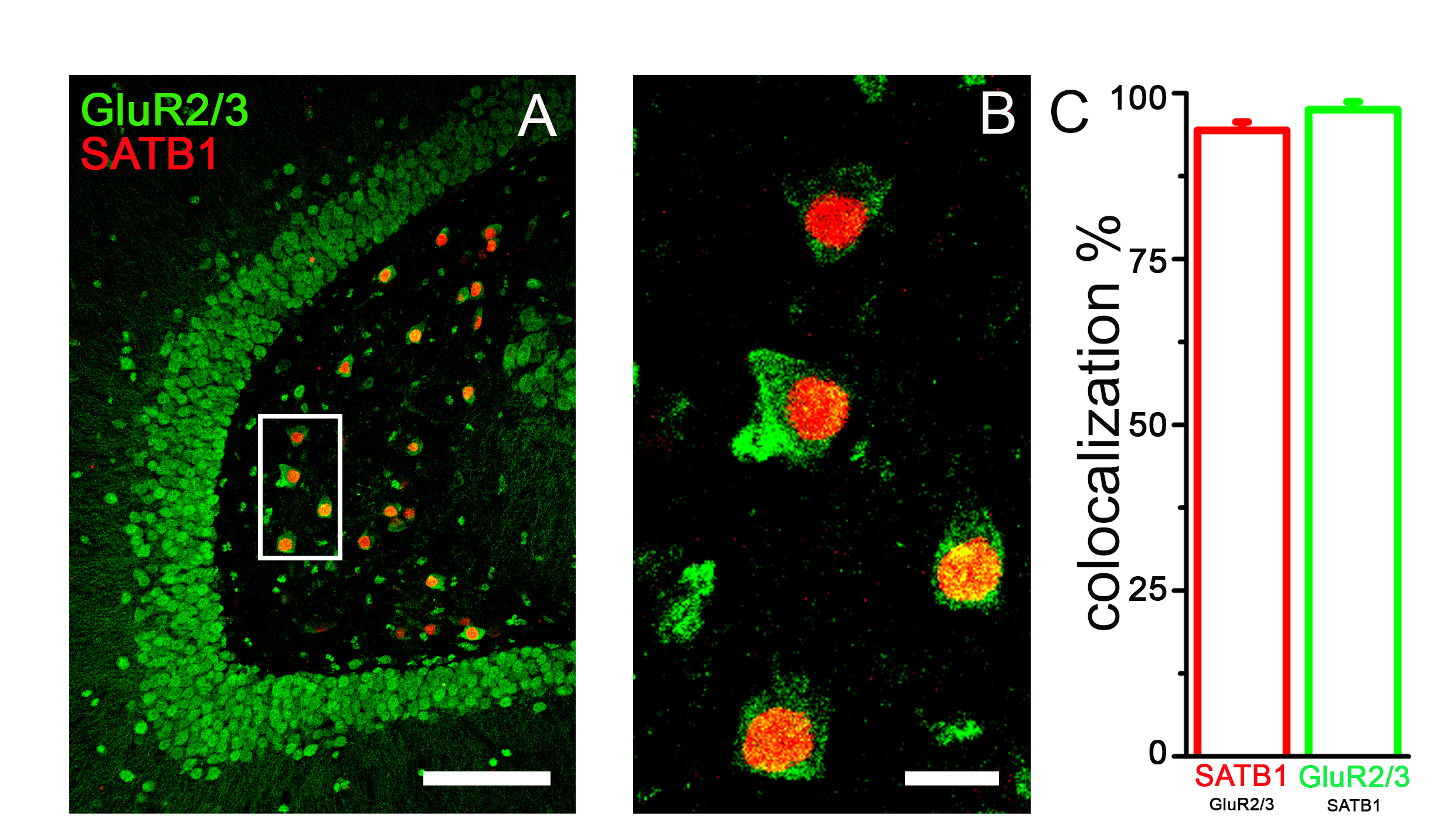

Supplement: Supplementary file 1 — Additional file 1. SATB1 and Glur2/3 are colocalized in the hilus. Representative 10xand 60xconfocal images of double immunofluorescence staining for SATB1and GLuR2/3.Left, percentage of SATB1 positive neurons that express GLuR2/3. Right, percentage of GLuR2/3 positive neurons that express SATB1. n = 211 neurons from 2 mice. Scale bar 100 μmand 5 μm. [file 12915_2023_1604_MOESM1_ESM.jpg]

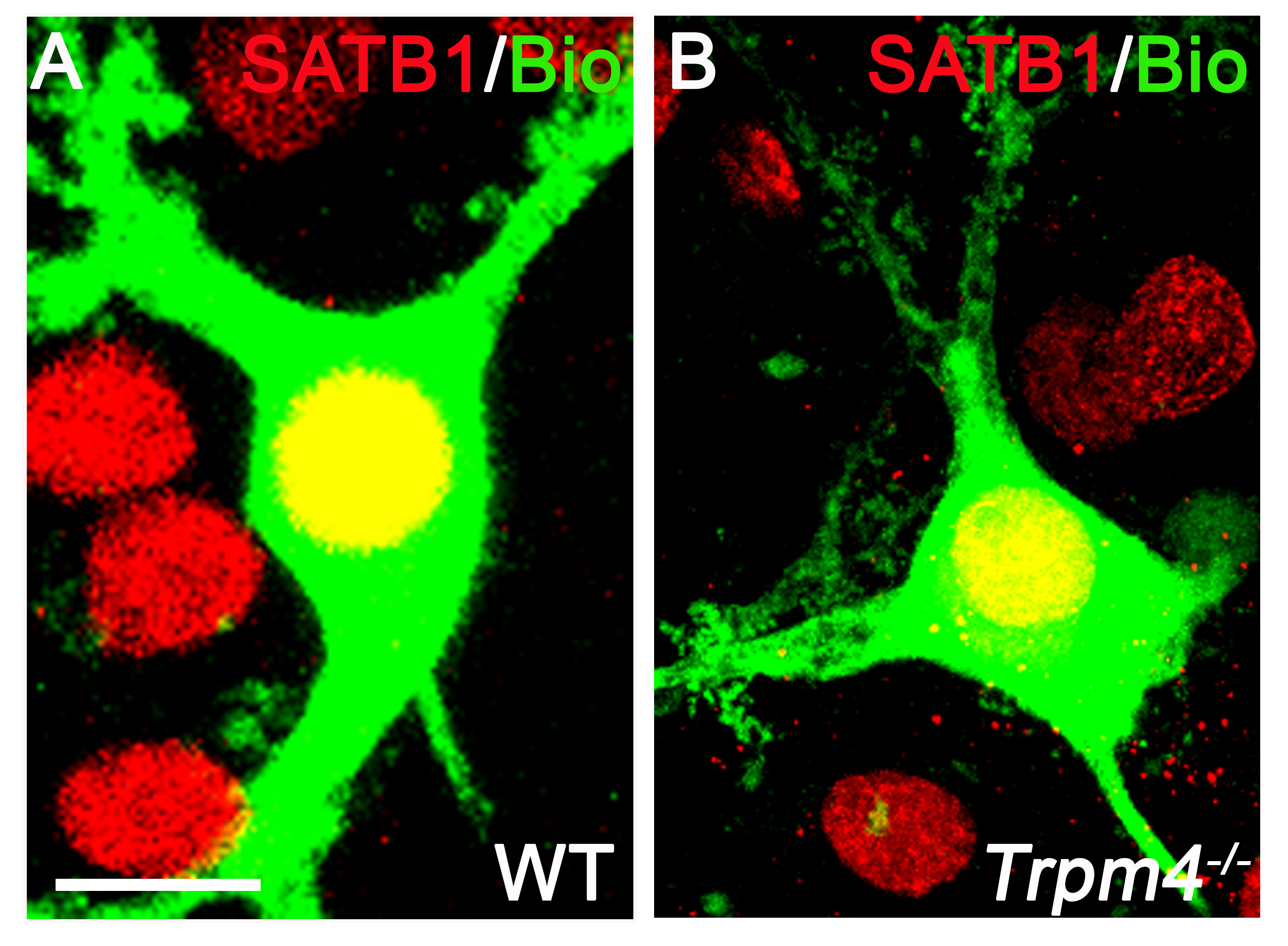

Supplement: Supplementary file 2 — Additional file 2. Patched neurons in the hilus are SATB1 positive. Representative confocal images of biocytinfilled WTand Trpm4−/−MCs counterstained with SATB1. Note that the biocytin filled cells are also SATB1 positive. Scale bar 5 μm. Image of WT neuronwas modified from previous publication [6]. [file 12915_2023_1604_MOESM2_ESM.jpg]

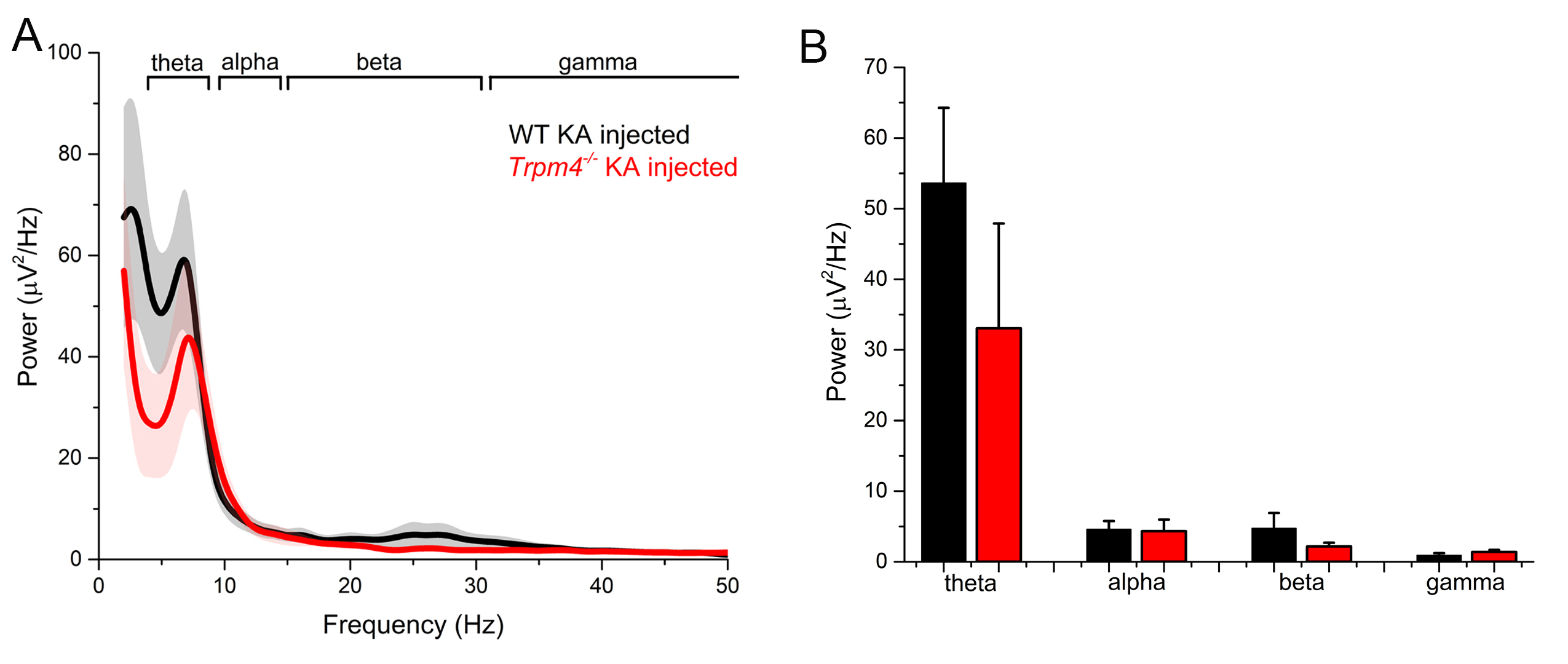

Supplement: Supplementary file 3 — Additional file 3. Power spectrum of epileptic WT and Trpm4−/− mice is not different.Power spectral density plot of epileptic WTand Trpm4−/−mice during exploration.Statistics showing theta, alpha, betaand gammapower in epileptic WTand Trpm4−/−mice. n = 6 for WT and 6 for Trpm4−/− mice. [file 12915_2023_1604_MOESM3_ESM.jpg]
