# Supplementary material for: TRPM4 regulates hilar mossy cell loss in temporal lobe epilepsy
Source: BMC Biol. 2023 Apr 26;21:96. doi: 10.1186/s12915-023-01604-3 (PMC10134545; doi:10.1186/s12915-023-01604-3)
Supplement: Supplementary file 5 — Additional file 5. Summary of statistics. [file 12915_2023_1604_MOESM5_ESM.docx]

**Summary of statistics:**

|  |  |  |  |  |  |
| --- | --- | --- | --- | --- | --- |
|  | **n number** | **mean** | **sem** | **test** | **p value** |
| Figure 2 A | WT=17  *Trpm4^-/-^*=13 | WT=-2.5  *Trpm4^-/-^*=-4.34 | WT=0.2  *Trpm4^-/-^*=0.64 | Two sample  T-test | 0.0049 |
| Figure 2 B | WT=19  *Trpm4^-/-^*=21 | WT=5.0  *Trpm4^-/-^*=4.0 | WT=0.4  *Trpm4^-/-^*=0.23 | Two sample  T-test | 0.03 |
| Figure 2 C | WT=20  *Trpm4^-/-^*=19 | WT=1.3  *Trpm4^-/-^*=0.5 | WT=0.2  *Trpm4^-/-^*=0.1 | Two sample  T-test | 0.0019 |
| Figure 2 D | WT=7  *Trpm4^-/-^*=6 | n.a. | n.a. | Fisher Exact Test | 0.029 |
|  |  |  |  |  |  |
| Figure 3 E | WT=9 | ACSF=1.4  9-phen.=0.44  Wash=1.3 | ACSF=0.2  9-phen.=0.1  Wash=0.2 | One-way Anova, Tukey’s post hoc | ACSF vs 9-phen=0.006 |
| Figure 3 D | WTsaline=3 WTKA=9  *Trpm4^-/-^*saline=3 *Trpm4^-/-^*KA=7 | WTsaline=100 WTKA=19.0 *Trpm4^-/-^*saline=91.8  *Trpm4^-/-^*KA=54 | WTsaline=5.15 WTKA=5  *Trpm4^-/-^*saline=4.2  *Trpm4^-/-^*KA=4 | One-way Anova, Tukey’s post hoc | Wtsaline vs WTKA= 9.5123E-7 *Trpm4^-/-^*saline vs *Trpm4^-/-^*KA=0.0025 WTKA vs *Trpm4^-/-^*KA=0.00025 |
| Figure 3 E | WTsaline=3 WTKA=8  *Trpm4^-/-^*saline=3 *Trpm4^-/-^*KA=7 | WTsaline=97.15 WTKA=64.62 *Trpm4^-/-^*saline=95.1  Trpm4-/-KA=77.68 | WTsaline=1.43 WTKA=11.34 *Trpm4^-/-^*saline=1.7  *Trpm4^-/-^*KA=4.08 | One-way Anova, Tukey’s post hoc | Wtsaline vs WTKA= 0.15  *Trpm4^-/-^*saline vs *Trpm4^-/-^*KA=0.65 WTKA vs *Trpm4^-/-^*KA=0.65 |
| Figure 4 D | WT=8  *Trpm4^-/-^*=7 | WT=66  *Trpm4^-/-^*=24.45 | WT=13  *Trpm4^-/-^*=9.1 | Mann-Whitney Test | 0.017 |
| Figure 4 E | WT=10  *Trpm4^-/-^*=10 | WT=7.3  *Trpm4^-/-^*=1.5 | WT=1.84  *Trpm4^-/-^*=0.74 | Mann-Whitney Test | 0.032 |
| Figure 4 F | WT=10  *Trpm4^-/-^*=10 | WT=1186  *Trpm4^-/-^*=336.5 | WT=410  *Trpm4^-/-^*=231.13 | Mann-Whitney Test | 0.044 |
| Figure 4 G | WT=10  *Trpm4^-/-^*=8 | WT=1.07  *Trpm4^-/-^*=0.85 | WT=0.13  *Trpm4^-/-^*=0.1 | Two sample  T-test | 0.22 |
| Figure 5 C | WTsaline=8 WTKA=9  *Trpm4^-/-^*saline=9 *Trpm4^-/-^*KA=8 | WTsaline=0.66 WTKA=0.45  *Trpm4^-/-^*saline=0.65  *Trpm4^-/-^*KA=0.54 | WTsaline=0.13 WTKA=0.05  *Trpm4^-/-^*saline=0.16  *Trpm4^-/-^*KA=0.13 | Kruskal-Wallis test | Wtsaline vs WTKA= 0.002  *Trpm4^-/-^*saline vs *Trpm4^-/-^*KA=0.11 |
| Figure 5 D | WTsaline=8 WTKA=9  *Trpm4^-/-^*saline=9 *Trpm4^-/-^*KA=8 | WTsaline=30.87 WTKA=37.44 Trpm4^-/-^_saline_=34.33 Trpm4^-/-^KA=32.87 | WTsaline=11.87 WTKA=16.69 *Trpm4^-/-^*saline=20.8  *Trpm4^-/-^*KA=8.8 | One-way Anova, Tukey’s post hoc | Wtsaline vs WTKA= 0.82 *Trpm4^-/-^*saline vs *Trpm4^-/-^*KA=0.99 |
| Figure 5 F | WTsaline=5 WTKA=5  *Trpm4^-/-^*saline=5 *Trpm4^-/-^*KA=5 | WTsaline=0.719 WTKA=0.671 Trpm4^-/-^_saline_=0.657 *Trpm4^-/-^*KA=0.736 | WTsaline=0.09 WTKA=0.14  *Trpm4^-/-^*saline=0.12  *Trpm4^-/-^*KA=0.13 | One-way Anova, Tukey’s post hoc | Wtsaline vs WTKA= 0.93 *Trpm4^-/-^*saline vs *Trpm4^-/-^*KA=0.75 |
| Figure 5 G | WTsaline=5 WTKA=5  *Trpm4^-/-^*saline=5 *Trpm4^-/-^*KA=5 | WTsaline=17.8 WTKA=17.4  Trpm4^-/-^_saline_=14.6  *Trpm4^-/-^*KA=12 | WTsaline=11.27 WTKA=7.43  *Trpm4^-/-^*saline=3.5 *Trpm4^-/-^*KA=5.04 | One-way Anova, Tukey’s post hoc | Wtsaline vs WTKA= 0.99 *Trpm4^-/-^*saline vs *Trpm4^-/-^*KA=0.94 |
